# Glycosylated RNAs in extracellular vesicles: a new layer of RNA sorting and intercellular communication

**DOI:** 10.20517/evcna.2026.32

**Published:** 2026-06-09

**Authors:** Zhelin Wu, Zhijian Cai

**Affiliations:** ^1^Institute of Immunology and Department of Orthopaedics of the Second Affiliated Hospital, Zhejiang University School of Medicine, Hangzhou 310058, Zhejiang, China.; ^2^Liangzhu Laboratory, Zhejiang University School of Medicine, Hangzhou 311121, Zhejiang, China.

## MAIN TEXT

The selective sorting of specific RNAs into exosomes remains a central, unresolved question in extracellular RNA biology. Previous studies have largely emphasized RNA-binding proteins and sequence motifs as determinants of RNA sorting, while emerging evidence suggests that RNA chemical modifications and cellular context may also play important roles in shaping extracellular vesicle (EV) RNA composition^[1]^. However, whether chemical modifications of RNA itself can act as intrinsic sorting signals has remained unclear. Recent advances in the identification and characterization of glycosylated RNAs (glycoRNAs) have begun to reveal their broader biological relevance beyond their initial discovery^[2]^.

A recent study published in Nature Cell Biology establishes a novel conceptual link between RNA chemistry and intercellular communication via extracellular vesicles, specifically highlighting the role of RNA glycosylation^[3]^. Using metabolic labeling with the azide-modified sugar analog Ac4GalNAz^[4]^, the authors enriched glycoRNAs from living cells and uncovered a striking distribution pattern: glycoRNAs were not detected at the plasma membrane or in the nucleus, but were instead enriched in membrane-associated cytoplasmic compartments and within the lumen of exosomes. This finding contrasts with earlier reports describing cell surface-associated glycoRNAs^[5]^, suggesting that glycoRNAs may comprise functionally distinct subpopulations defined by their subcellular localization: (1) ‌cell-surface glycoRNAs‌, which are displayed on the outer leaflet of the plasma membrane and likely mediate direct cell-cell interactions; (2) ‌exosome-luminal glycoRNAs‌, the focus of this study, which are actively sorted into the vesicle lumen via the Endosomal Sorting Complex Required for Transport (ESCRT) pathway and packaged for protected, long-range delivery; and (3) a putative third class of ‌exosome-surface-associated glycoRNAs‌ that may be transiently or peripherally associated with the exosomal membrane exterior and whose roles remain to be resolved. This spatial partitioning implies a functional divergence where surface-exposed glycoRNAs could play immediate roles in immune recognition or cell adhesion, while luminal glycoRNAs are specialized for intercellular communication.

While the metabolic labeling and click chemistry approach provides a powerful strategy for glycoRNA enrichment, a methodological caveat warrants attention. Glycoprotein co-purification may contribute to glycan signals in glycoRNA preparations. Although RNase and proteinase K treatments were applied to reduce protein contamination, residual glycoproteins or glycan-binding proteins cannot be fully excluded. Future validation using stringent detergent washes, mass spectrometry-based linkage characterization, glycosidase digestion, or orthogonal sugar analog labeling would help confirm direct RNA-glycan conjugation.

Notably, the identified glycoRNAs are predominantly small non-coding RNAs (< 200 nucleotides), including snoRNAs, snRNAs, and Y RNAs. This size bias raises important mechanistic questions. One possibility is that short RNAs are more compatible with vesicle loading machinery or preferentially recognized by RNA-binding adaptors involved in cargo selection. Alternatively, glycosylation itself may be structurally more accessible on compact RNA species, thereby favoring smaller transcripts^[1]^. In addition, glycoRNAs display resistance to RNase A digestion, suggesting that glycosylation may enhance RNA stability and facilitate their persistence in the extracellular environment.

Mechanistically, disruption of exosome biogenesis, either through genetic inhibition of ESCRT components such as HRS^[6,7]^ or pharmacological blockade using GW4869^[8]^ and manumycin A^[9]^, led to a marked reduction of glycoRNAs in exosomes, accompanied by their intracellular accumulation. These findings support a model in which glycoRNAs are actively sorted into exosomes via canonical vesicle biogenesis pathways.

Intriguingly, the study also points to a functional interplay between RNA and protein glycosylation networks. Perturbation of protein glycosylation pathways, including knockdown of GALE or GNE^[10]^ and inhibition of oligosaccharyltransferase activity^[11]^, significantly reduced glycoRNA levels. This observation suggests that RNA and protein glycosylation may share common metabolic substrates and regulatory pathways. Such coordination raises the possibility that cellular metabolic states globally influence glycosylation-dependent cargo selection, given that nucleotide sugar metabolism and glycosylation pathways are tightly integrated within cellular networks^[12]^.

Beyond mechanistic insight, glycoRNAs are capable of intercellular transfer via exosomes. Exosomes derived from labeled donor cells are efficiently taken up by recipient cells, enabling rapid delivery of glycoRNAs. Moreover, the composition of exosomal glycoRNAs dynamically changes during stem cell differentiation, suggesting that RNA glycosylation and vesicle-mediated sorting are responsive to cellular state and may participate in developmental or adaptive processes.

The existence of glycoRNAs in distinct subcellular compartments further raises important questions regarding their biological functions. Surface-exposed glycoRNAs may participate in immune recognition or cell-cell interactions, whereas exosome-associated glycoRNAs may mediate long-range intercellular communication. Differences in glycan structures, RNA species, and interacting partners may further diversify their functional roles across cellular contexts.

## CONCLUSION AND FUTURE PERSPECTIVES

This study identifies RNA glycosylation as a novel molecular feature potentially associated with selective RNA sorting into exosomes, thereby adding a new layer of regulation to extracellular RNA biology. By linking RNA chemical modification to vesicle-mediated communication, these findings expand the current framework of RNA sorting beyond protein-centric models. However, while these findings strongly associate RNA glycosylation with exosomal enrichment, definitive proof that glycosylation per se serves as an intrinsic and sufficient sorting signal awaits future functional studies that directly manipulate specific glycoRNA structures.

Several key questions remain to be addressed in future research. First, the ‌exact chemical linkage‌ between glycans and RNAs, whether N-linked, O-linked, or through other covalent bonds, remains to be precisely elucidated. Second, the ‌enzymatic machinery‌ responsible for catalyzing RNA glycosylation, including the identification of the specific glycosyltransferases and their substrate recognition mechanisms, is largely unknown. Third, beyond the sorting process, the ‌biological functions‌ of glycoRNAs following exosomal transfer to recipient cells are a critical frontier. It is imperative to determine whether and how glycoRNAs influence recipient cell physiology, such as modulating immune responses, altering signaling pathways, or contributing to disease progression. Furthermore, the determinants of substrate specificity, particularly the preference for small non-coding RNAs, require further investigation. Future studies should also explore how metabolic and signaling networks coordinate RNA and protein glycosylation, particularly in the context of glycosylation pathway integration and cellular nutrient states.

**Figure 1:**
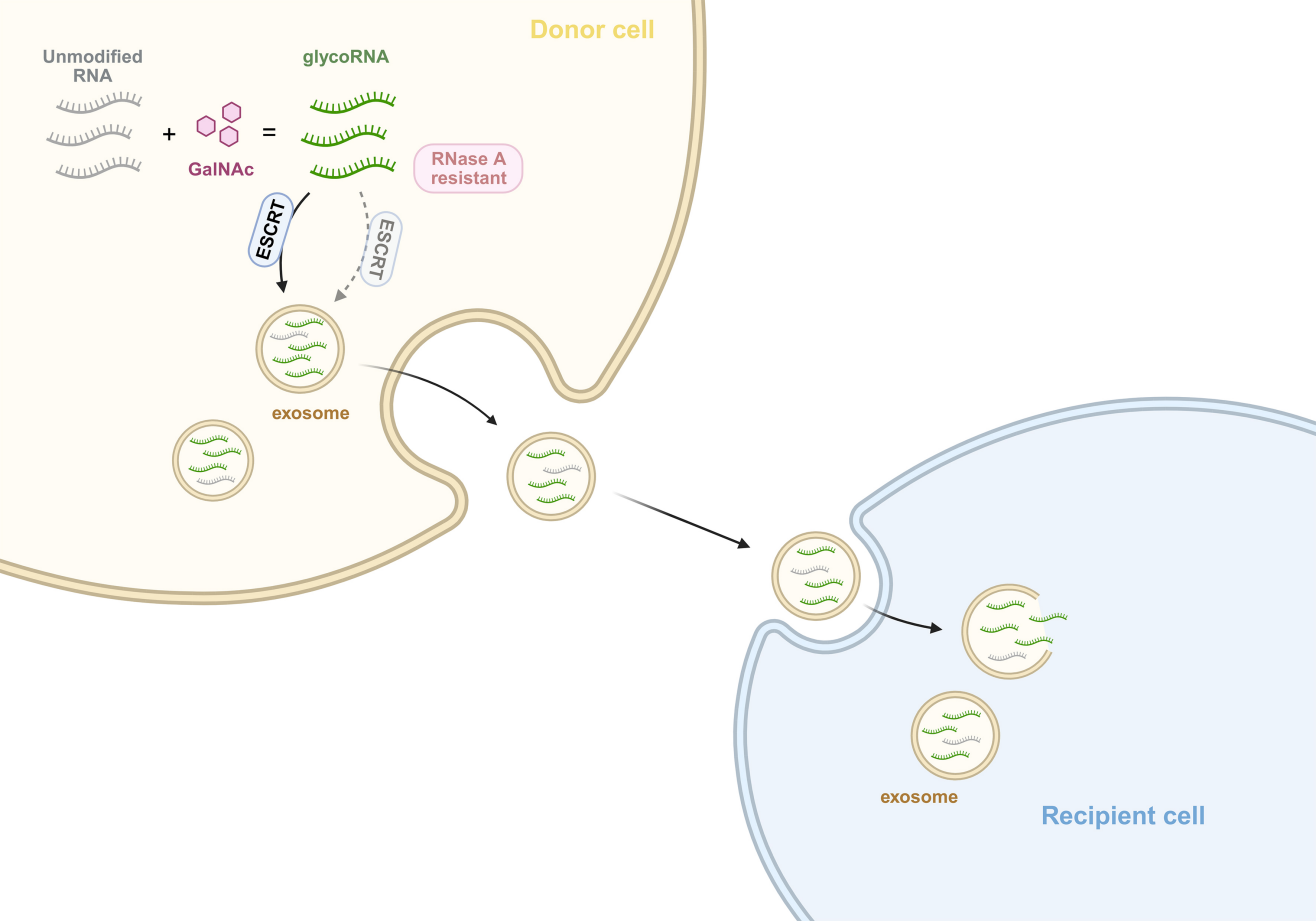
Glycosylation regulates exosomal RNA sorting and intercellular transfer. In donor cells, a subset of small non-coding RNAs undergoes GalNAc-dependent glycosylation, which is associated with their preferential enrichment into exosomes during biogenesis. This specific cargo-loading process depends on canonical exosome biogenesis pathways, including both ESCRT-dependent and ESCRT-independent mechanisms. Subsequently, these glycoRNAs are encapsulated within the exosomal lumen. Exosomes containing glycoRNAs can be released and taken up by recipient cells, facilitating the intercellular transfer of glycoRNAs. Created in BioRender. Wu, Z. (2026) https://BioRender.com/cv1j34x. GalNAc: N-acetylgalactosamine; RNA: ribonucleic acid; ESCRT: endosomal sorting complex required for transport; glycoRNA: glycosylated RNA.

From a translational perspective, glycoRNAs may represent a new class of biomarkers or therapeutic targets in diseases involving extracellular vesicles. Overall, these findings open new avenues at the intersection of RNA biology, glycobiology, and intercellular communication [‌Figure 1‌].

## DECLARATIONS

### AI and AI-assisted tools statement

During the preparation of this manuscript, the AI tool ChatGPT (version 1.2026.051, released 2026-3) was used solely for grammar revision. The tool did not influence the study design, data collection, analysis, interpretation, or the scientific content of the work. All authors take full responsibility for the accuracy, integrity, and final content of the manuscript.

### Copyright

© The Author(s) 2026.
